# *In Vivo* Fitness Adaptations of Colistin-Resistant Acinetobacter baumannii Isolates to Oxidative Stress

**DOI:** 10.1128/AAC.00598-16

**Published:** 2017-02-23

**Authors:** Crystal L. Jones, Shweta S. Singh, Yonas Alamneh, Leila G. Casella, Robert K. Ernst, Emil P. Lesho, Paige E. Waterman, Daniel V. Zurawski

**Affiliations:** a Wound Infections Department, Walter Reed Army Institute of Research, Silver Spring, Maryland, USA; b Department of Microbial Pathogenesis, University of Maryland School of Dentistry, Baltimore, Maryland, USA; c Multidrug-resistant Organism Repository and Surveillance Network, Walter Reed Army Institute of Research, Silver Spring, Maryland, USA

**Keywords:** Acinetobacter, ESKAPE, catalase, fitness, mass spectrometry, mouse model, serial isolation, virulence

## Abstract

The loss of fitness in colistin-resistant (CR) Acinetobacter baumannii was investigated using longitudinal isolates from the same patient. Early CR isolates were outcompeted by late CR isolates for growth in broth and survival in the lungs of mice. Fitness loss was associated with an increased susceptibility to oxidative stress since early CR strains had reduced *in vitro* survival in the presence of hydrogen peroxide and decreased catalase activity compared to that of late CR and colistin-susceptible (CS) strains.

## TEXT

Colistin resistance (CR) in Acinetobacter baumannii is due to the addition of phosphoethanolamine (1, 2) and galactosamine to the lipid A portion of the lipopolysaccharide (LPS) (3) and is mediated by the upregulation of the PmrAB two-component system (4). CR leads to the loss of fitness and virulence in laboratory-derived and clinical isolates of A. baumannii (5–7), suggesting that adapting to colistin stress may be detrimental to the survival of this pathogen. However, these studies were only evaluated in a single CR isolate and, thus, do not take into account the potential adaptations made by bacteria throughout the course of infection.

**FIG 1:**
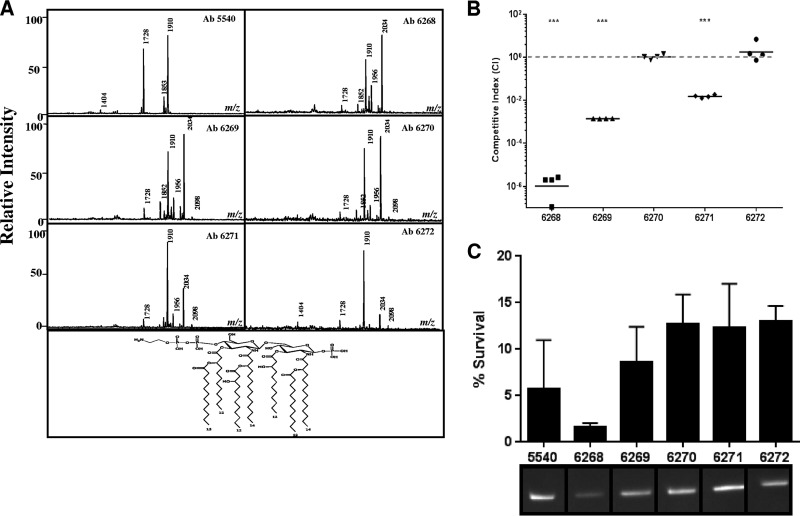
Fitness loss in colistin-resistant A. baumannii was due to enhanced susceptibility to oxidative stress and a defect in catalase activity. (A) Mass spectra of lipid A of A. baumannii clinical isolates. The peak at 2,034 *m/z* represents phosphoethanolamine. (B) A. baumannii clinical isolates competed for growth in LB for 24 h. Each symbol represents a single replicate. Data are representative of those from three independent experiments. (C) The survival of CR A. baumannii clinical isolates in the presence of 0.001% hydrogen peroxide was measured after 2 h. The percent survival was assessed as the CFU ratio of treated and untreated samples. Total catalase activity was quantified using in-gel catalase assays. Statistical analyses of competition experiments (one sample *t* test) were performed using GraphPad Prism. ***, *P* < 0.0005.

In this study, our goal was to further investigate the association between CR and fitness loss as it developed *in vivo* by performing a comparative analysis of five longitudinal CR A. baumannii strains that were serially obtained from the same patient following colistin therapy. Briefly, the patient was hospitalized in an intensive care unit or surgical ward after sustaining severe trauma injuries in combat operations outside of the United States (8). The patient received antibiotics for complicated infections during an initial hospitalization in Afghanistan, transient hospitalization in Germany, and definitive hospitalization in the United States. The bacterial isolates used in this study (parental, colistin-susceptible [CS] MRSN 5540 and CR clinical isolates MRSN 6268, MRSN 6269, MRSN 6270, MRSN 6271, and MRSN 6272) were obtained from successive cultures from the urinary or respiratory tract (8). CR in all of the strains was due to a point mutation in *pmrA* and/or *pmrB* (8) that resulted in the addition of phosphoethanolamine to the lipid A portion of the LPS (Fig. 1A).

Fitness loss was measured using competition assays. For *in vitro* experiments, 100 μl of bacterial inoculum was grown in 20 ml of lysogeny broth (LB) at 37°C for 24 h, and bacteria were plated as described above. Early CR isolates were also outcompeted by the CS isolate for growth in LB after 24 h, with MRSN 6268 being the least fit strain. Late CR isolates showed a steady increase in fitness, culminating with the final isolate MRSN 6272 having a competitive index (CI) of >1.0 (Fig. 1B). There was no significant difference in SDS susceptibilities of CR isolates (data not shown), indicating that the loss of fitness was not due to an inherent loss of membrane integrity.

The isolates used in this study were previously exposed to colistin during infection, which can kill A. baumannii through the production of hydroxyl radicals (9, 10). To address whether oxidative stress contributed to the fitness defect in CR isolates, we grew bacteria in the presence or absence of 0.001% hydrogen peroxide for 2 h and the percent survival rate was calculated. Early CR isolate MRSN 6268 had an ∼2% survival rate in the presence of hydrogen peroxide, while late isolates MRSN 6270 to 6272 had an ∼12% survival rate (Fig. 1C). Catalases play important roles in protecting bacteria from damage caused by reactive oxygen species by hydrolyzing hydrogen peroxide (11). Therefore, an in-gel catalase assay was performed as previously described (12) to determine whether the increased susceptibility of early isolates to oxidative stress was due to altered total catalase activity. Indeed, early CR isolate MRSN 6268 exhibited a reduced level of catalase activity compared to other CR isolates (Fig. 1C), suggesting that the increase in susceptibility to oxidative stress in early CR isolates was due to an inability to break down hydroxyl radicals. Sun et al. recently showed that hydrogen peroxide resistance in A. baumannii was mediated by KatG and KatE (13), which are also conserved in these isolates. It is possible that the expression of catalase genes in CR isolates is altered, and further characterization of these genes is needed to determine whether they play a role in decreased fitness.

**FIG 2:**
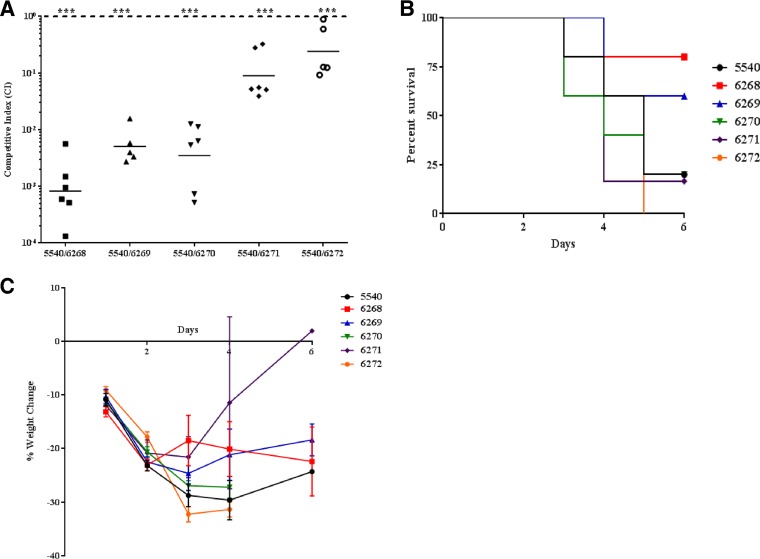
*In vivo* fitness adaption of colistin-resistant A. baumannii. (A) Colistin-resistant A. baumannii clinical isolates were competed for growth in the lungs of mice for 48 h. Data are combined from two independent experiments, and each symbol represents a single animal. (B) Survival curves of mice (*n* = 5) intranasally infected with 1 × 10^8^ CFU of A. baumannii. (C) Weight change of mice infected intranasally with 1 × 10^8^ CFU of A. baumannii. Data are representative of two independent animal experiments. Statistical analysis of competition experiments (one-sample *t* test) was performed using GraphPad Prism. ***, *P* < 0.0005.

Using a neutropenic mouse model of lung infection, we investigated the *in vivo* fitness of longitudinal isolates. Briefly, cyclophosphamide-treated BALB/c mice were intranasally infected with 5.0 × 10^6^ CFU of an inoculum containing a 1:1 ratio of the CS isolate and the indicated CR strain was suspended in sterile phosphate-buffered saline (PBS). The lungs were collected at 48 h, and homogenates were plated on lysogeny broth (LB) agar with and without colistin sulfate. The competitive index (CI) was calculated using the following formula: CI = (mutant CFU_output_/wild-type CFU_output_)/(mutant CFU_input_/wild-type CFU_input_) (14). Interestingly, early CR isolates were outcompeted by the CS strain for growth in the lungs of mice at 48 h; however, there was a steady increase in fitness of A. baumannii clinical isolates, as they were isolated from the patient longitudinally (Fig. 2A). To determine whether *in vivo* adaptations of latter CR isolates correlated with increased virulence, we performed survival studies. Mice were intranasally infected with 1 × 10^8^ CFU of each strain and monitored for signs of morbidity. We found that mice infected with the CS strain had susceptibilities similar to those of mice infected with late CR isolates (Fig. 2B). Mice infected with late CR isolates had a 0% to 25% survival rate, which is in stark contrast to the 60% to 80% survival rates of mice infected with early CR isolates. Furthermore, mice that died from A. baumannii infection exhibited a >25% decrease in body weight (Fig. 2C). These data showed that increased virulence in late CR isolates correlated with increased *in vivo* fitness, suggesting that A. baumannii may compensate for the possible growth defect associated with developing colistin resistance (Fig. 2B). It should be noted that virulence in this model is relative since a high dose of bacterial inoculum was required to establish infection in mice that were immunosuppressed using cyclophosphamide, a drug that causes transient leukopenia in this mouse model of infection. All of the research presented was conducted in compliance with the Animal Welfare Act and other federal statutes and regulations relating to animals and experiments involving animals and adheres to principles stated in the *Guide for the Care and Use of Laboratory Animals* (15).

Colistin resistance has been associated with *in vitro* growth defects in A. baumannii. Therefore, we performed growth curves of A. baumannii in nutrient-rich media (see Fig. S1 in the supplemental material) and found that MRSN 6268 and MRSN 6271 had significant defects in growth compared to MRSN 5540 up to 12 h, suggesting that the observations associated with decreased fitness and virulence in MRSN 6268 may be due to a generalized growth defect. Interestingly, MRSN 6271 had increased virulence and fitness despite the *in vitro* growth defect. We speculate that the growth defect, while an important contributing factor, may not impact the outcome of infection since all strains were able to grow equally at 12 h. To address the contradicting *in vitro* growth defect, we measured bacterial burden in the lungs of mice infected with 5 × 10^6^ CFU at 24 h postinfection. As previously mentioned, the absence of an intact cellular immune system in neutropenic mice allows for the observation of inherent differences in growth of study isolates. There was no difference in bacterial burden in the lungs of mice infected with study isolates after 24 h, suggesting that the *in vitro* growth defect observed in A. baumannii may not correlate with the ability of bacteria to grow in the host.

Our findings further characterize the phenotype of CR A. baumannii clinical isolates *in vivo* by showing that CR isolates underwent an initial loss in fitness and virulence, partially due to an inability to cope with oxidative stress. This fitness and virulence defect was compensated for in late CR isolates, and adaptive mutations likely attribute increased fitness and virulence. The absence of single nucleotide polymorphisms (SNPs) in virulence genes of CR isolates, as revealed by whole-genome sequencing (data not shown), suggests that bacterial adaptions to increase fitness and virulence may be due to posttranslational modifications or physiological changes. Taken together, these findings highlight an area of weakness for this emerging superbug that can be potentially exploited by drug intervention.

## Supplementary Material

Supplemental material
