# Supplementary material for: In Vivo Fitness Adaptations of Colistin-Resistant Acinetobacter baumannii Isolates to Oxidative Stress
Source: Antimicrob Agents Chemother. 2017 Feb 23;61(3):e00598-16. doi: 10.1128/AAC.00598-16 (PMC5328574; doi:10.1128/AAC.00598-16)
Supplement: Supplemental material [file supp_61_3_e00598-16__index.html]

In Vivo Fitness Adaptations of Colistin-Resistant Acinetobacter baumannii Isolates to Oxidative Stress — Supplemental material 

# *In Vivo* Fitness Adaptations of Colistin-Resistant Acinetobacter baumannii Isolates to Oxidative Stress

## Supplemental material

- Supplemental file 1 -

  Figure S1

  PDF, 55K
