# Supplementary material for: In Vivo Fitness Adaptations of Colistin-Resistant Acinetobacter baumannii Isolates to Oxidative Stress
Source: Antimicrob Agents Chemother. 2017 Feb 23;61(3):e00598-16. doi: 10.1128/AAC.00598-16 (PMC5328574; doi:10.1128/AAC.00598-16)
Supplement: Supplemental material [file AAC.00598-16_zac003175936s1.pdf]

Supplemental Figure 1

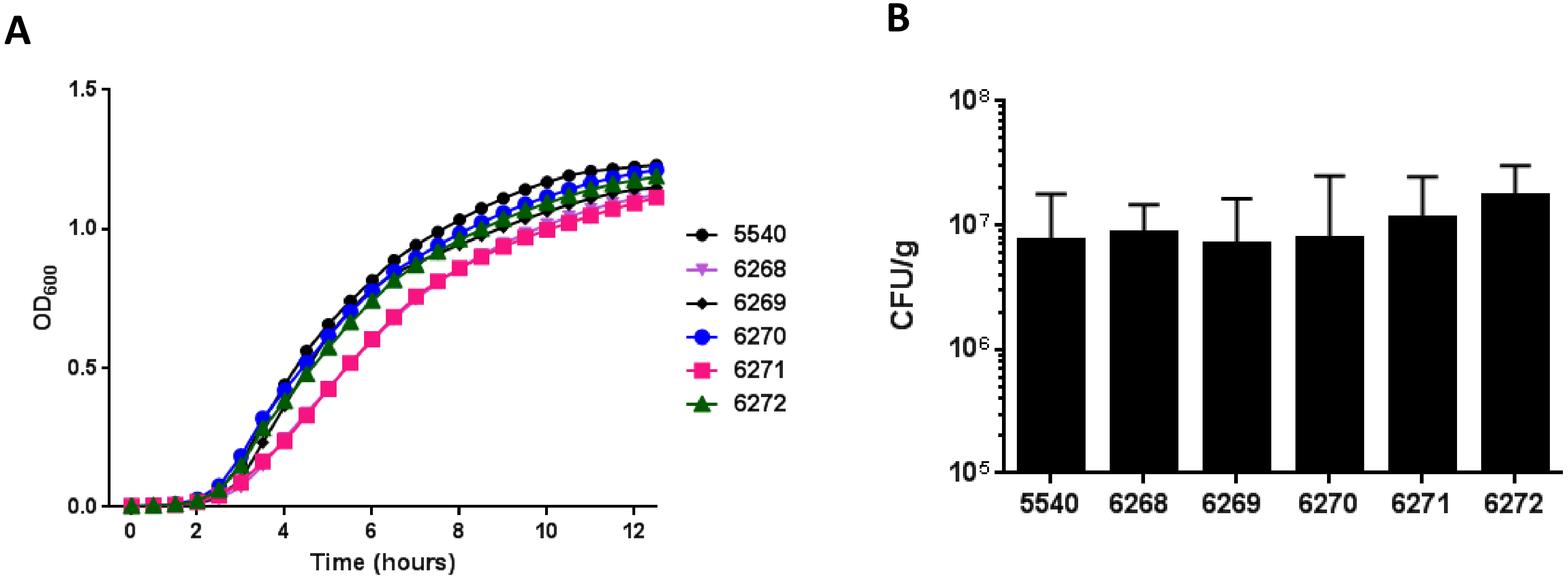

Growth of *A. baumannii* (A) Growth curves of *A. baumannii* isolates in LB broth and (B) in the lungs of neutropenic mice at 24h post-infection. Mice were infected intranasally with 5.0 x 10^6 CFU of bacteria suspended in sterile PBS. Statisical analysis of growth curve was performed using ANOVA with Dunnett's test.
